# Dental Anxiety and Associated Demographic and Dental Attendance Factors: A Cross-Sectional Study Using the Modified Dental Anxiety Scale

**DOI:** 10.3390/healthcare14193190

**Published:** 2026-09-28

**Authors:** Abdurrahman Yalçın, Makbule Taşyürek, Özkan Adıgüzel

**Affiliations:** 1 Department of Restorative Dentistry, Faculty of Dentistry, Dicle University, Diyarbakir 21280, Turkey; 2 Department of Endodontics, Faculty of Dentistry, Dicle University, Diyarbakir 21280, Turkey

**Keywords:** dental anxiety, Modified Dental Anxiety Scale, MDAS, dental attendance, Firth logistic regression, cross-sectional study

## Abstract

Background: Dental anxiety is a common barrier to dental care and may influence treatment-seeking behavior and regular attendance. This study aimed to evaluate dental anxiety using the Modified Dental Anxiety Scale (MDAS), examine its distribution across demographic and dental-care characteristics, and identify factors independently associated with high dental anxiety. Methods: This cross-sectional study included 1005 participants aged ≥ 16 years attending dental clinics. Dental anxiety was assessed using the five-item MDAS, with scores ≥19 indicating high dental anxiety. Group differences in MDAS scores were evaluated using non-parametric tests, and internal consistency was assessed using Cronbach’s alpha. Associations with high dental anxiety were examined using categorical analyses and multivariable Firth-penalized logistic regression. Results: The median MDAS score was 11 (IQR, 7–15), and 49 participants (4.9%) had high dental anxiety. MDAS scores differed significantly according to gender, age group, education level, dental attendance frequency, reason for dental visit, and history of post-treatment complications (all *p* < 0.05). Anxiety differed significantly across the five MDAS items (Friedman χ^2^(4) = 624.477, *p* < 0.001), with anticipation of a dental visit producing the highest mean item score. The MDAS demonstrated good internal consistency (Cronbach’s α = 0.812). In multivariable Firth regression, gender, age group, dental attendance frequency, and primary reason for dental visit showed significant overall associations with high dental anxiety, whereas education level was not significantly associated after adjustment. Regular dental attendance was associated with lower odds of high dental anxiety. However, several category-specific estimates were highly imprecise because of sparse and clustered high-anxiety events, and sensitivity analysis indicated that some effect magnitudes were sensitive to model specification. Conclusions: Dental anxiety was generally low to moderate in this clinical sample, although a small subgroup met the threshold for high dental anxiety. Gender, age, dental attendance patterns, and reason for dental visit were associated with high dental anxiety. Regular dental attendance was associated with lower odds of high dental anxiety; however, the cross-sectional design precludes inference regarding the direction of this relationship. Brief anxiety screening and individualized communication may help identify and support patients with greater dental anxiety.

## 1. Introduction

Dental anxiety is a common psychological response to dental care, ranging from anticipatory apprehension to severe fear that may interfere with treatment. High levels of dental anxiety can negatively affect treatment tolerance, patient–dentist communication, and dental attendance behavior [1,2]. Anxious individuals may postpone or avoid routine dental care and seek treatment primarily when symptoms become severe, potentially contributing to a cycle of delayed attendance, deteriorating oral health, greater treatment needs, and further negative dental experiences [3,4,5].

Dental anxiety is influenced by multiple demographic and dental-related factors. Associations with gender, age, educational level, previous dental experiences, and patterns of dental attendance have been reported, although findings vary across populations [3,6]. Previous painful or adverse dental experiences and anxiety-provoking procedures, particularly local anesthetic injections, drilling, and invasive treatments, may further contribute to dental anxiety [7,8,9,10]. These findings highlight the importance of evaluating demographic characteristics together with previous dental experiences and treatment-seeking behaviors.

The Modified Dental Anxiety Scale (MDAS) is a brief and widely used five-item instrument assessing anxiety associated with an anticipated dental visit, waiting for treatment, dental drilling, scaling, and local anesthetic injection [11,12]. The scale provides a total score ranging from 5 to 25, with higher scores indicating greater dental anxiety. The MDAS has demonstrated satisfactory validity and reliability across different populations, including Turkish samples [12,13]. In addition to evaluating overall anxiety severity, an MDAS score ≥19 can be used to identify individuals with high dental anxiety, allowing factors associated with this clinically relevant subgroup to be investigated [14,15].

Although dental anxiety has been examined previously in relation to demographic characteristics and dental attendance, fewer studies have simultaneously evaluated continuous MDAS scores, clinically defined high dental anxiety, item-level response patterns, and multivariable associations within a large Turkish clinical sample. The present study therefore aimed not only to describe overall dental anxiety but also to examine how demographic characteristics, attendance patterns, and reasons for seeking dental care were associated with both the broader distribution of MDAS scores and the smaller subgroup meeting the established threshold for high dental anxiety.

Therefore, this study aimed to assess dental anxiety among patients presenting to the Faculty of Dentistry, Dicle University, and to investigate its associations with gender, age, educational level, place of residence, previous dental attendance, primary reason for dental visit, dental attendance frequency, and history of post-treatment complications. The study additionally evaluated the internal consistency and item-level characteristics of the MDAS and investigated factors independently associated with high dental anxiety (MDAS ≥19). The null hypothesis was that dental anxiety would not differ according to the demographic and dental-history characteristics evaluated.

## 2. Materials and Methods

### 2.1. Study Design, Setting, and Ethical Considerations

This cross-sectional, observational questionnaire-based study was conducted at the Faculty of Dentistry, Dicle University, to evaluate the level of dental anxiety and factors associated with dental anxiety among individuals presenting for dental care. The study protocol was approved by the Ethics Committee of the Faculty of Dentistry, Dicle University (approval date: 19 June 2025; decision no. 2025-22). The study was conducted in accordance with the principles of the Declaration of Helsinki. Before participation, all individuals were informed about the purpose and content of the study, the voluntary nature of participation, and the use of the collected data exclusively for scientific purposes. Written informed consent was obtained from all adult participants prior to enrollment. For participants aged 16–17 years, written informed consent was obtained from a parent or legal guardian. Participants were recruited using a convenience sampling approach among individuals presenting to the Faculty of Dentistry during the study period. Data collection was carried out between 1 July 2025 and 31 May 2026. The study protocol initially planned the recruitment of at least 1000 individuals. A total of 1005 participants with complete questionnaire data were ultimately included in the analysis.

### 2.2. Study Population and Eligibility Criteria

The study population consisted of individuals presenting to the Faculty of Dentistry, Dicle University. Participants were eligible if they were ≥16 years of age, voluntarily agreed to participate, and had sufficient cognitive and linguistic ability to understand and complete the questionnaire. Individuals with a known diagnosed psychiatric disorder were excluded to reduce potential confounding related to psychiatric conditions that could independently influence anxiety responses and questionnaire completion. Age was available in the analytic dataset only in predefined categories; therefore, the exact number of participants aged 16–17 years could not be determined from the analysis file. Participants aged ≥ 60 years were eligible for inclusion; however, none were represented in the recruited sample. Individuals were excluded if they were younger than 16 years, declined participation, had a cognitive or communication problem that could interfere with comprehension or completion of the questionnaire, reported a previously diagnosed psychiatric disorder, or returned an incomplete questionnaire. Following application of these criteria, complete data from 1005 participants were available for statistical analysis.

### 2.3. Sample Size Considerations

Before participant recruitment, an a priori sample-size calculation was performed using G*Power version 3.1.9.7 (Heinrich Heine University, Düsseldorf, Germany). Using the one-way fixed-effects omnibus ANOVA framework as an approximation for planned comparisons across multiple participant groups, with an effect size of f = 0.15, α = 0.05, statistical power of 95%, and seven groups, the minimum required total sample size was calculated as 938 participants. The study protocol nevertheless targeted the recruitment of at least 1000 participants to obtain a larger clinical sample and to improve representation across demographic and dental-care subgroups. The final sample of 1005 participants exceeded both the calculated minimum and the predefined recruitment target.

### 2.4. Data Collection and Study Variables

Data were collected from individuals presenting to all clinical departments of the Faculty of Dentistry, Dicle University, between 1 July 2025 and 31 May 2026. Participants were recruited using a convenience sampling approach and were not enrolled consecutively. Questionnaires were administered face-to-face in the clinical setting. Participation was voluntary, and written informed consent was obtained before completion of the questionnaire. Before participation, individuals were informed about the purpose and importance of the study, the voluntary nature of participation, and the use of the collected data exclusively for scientific purposes.

A total of 1200 eligible individuals were approached and invited to participate. Of these, 195 declined participation, and 1005 completed the questionnaire and were included in the final analysis, corresponding to a participation rate of 83.8%. The questionnaire consisted of the Modified Dental Anxiety Scale (MDAS) together with a structured form assessing demographic characteristics and dental-history variables. Completed questionnaires were reviewed for completeness before analysis, and all 1005 returned questionnaires contained complete data for the variables included in the study. No personally identifying information was collected. Questionnaires were distributed and collected by the dentist responsible for each clinical unit. Access to the completed questionnaires and study dataset was restricted to the principal investigator (first author), and the data were analyzed anonymously.

Data were collected using a structured questionnaire consisting of two components: (a) demographic and dental-history characteristics and (b) the MDAS. The demographic and dental-history section recorded gender, age group, educational level, place of residence, history of previous dental attendance, primary reason for the current dental visit, usual frequency of dental attendance, and history of complications following previous dental treatment. Age was recorded categorically as <20, 20–29, 30–39, 40–49, 50–59, or ≥60 years. Because no participants aged ≥ 60 years were represented in the final analytic dataset, age-group analyses comprised five categories (<20, 20–29, 30–39, 40–49, and 50–59 years).

Educational status was originally collected using eight categories: illiterate, literate without formal schooling, primary school, middle school, high school, associate degree, bachelor’s degree, and master’s degree. To provide analytically meaningful groups and avoid excessive fragmentation, educational status was subsequently grouped as literate or lower, primary/secondary education, and university or higher for inferential analyses. Place of residence was categorized as village, district, or city. Previous dental attendance and history of complications following dental treatment were recorded as binary variables (yes/no). Dental attendance frequency was categorized as first visit, attendance only when symptomatic, regular attendance every 6 months, or regular annual attendance. The primary reason for the dental visit was classified as extraction, filling, root canal treatment, scaling, prosthetic treatment, orthodontic treatment, or periodontal surgery. Because only three participants reported periodontal surgery as their primary reason for attendance, this category was retained for descriptive reporting but excluded from inferential analyses involving visit reason to avoid unstable estimates arising from the extremely small subgroup size. The complete study questionnaire, including all demographic and dental-history questions and their original response options, is provided in Supplementary Material S1.

### 2.5. Assessment of Dental Anxiety

Dental anxiety was assessed using the Modified Dental Anxiety Scale (MDAS) developed by Humphris et al. [16]. The MDAS is a five-item instrument designed to assess anticipatory and procedure-related dental anxiety across commonly encountered dental situations. The five items assess anxiety associated with: (a) anticipating a dental visit on the following day, (b) waiting for treatment in the dental clinic, (c) anticipating dental drilling, (d) anticipating scaling, and (e) receiving a local anesthetic injection. Each item is scored on a five-point ordinal scale ranging from 1 to 5, with higher scores indicating greater anxiety.

Individual item scores were summed to obtain the MDAS total score, with a theoretical range of 5–25. The total MDAS score was analyzed as a continuous/ordinal measure for comparisons of anxiety severity and was also dichotomized for analyses of high dental anxiety. Participants with an MDAS total score of ≥19 were classified as having high dental anxiety, whereas those with scores <19 were classified as not having high dental anxiety. This binary outcome was subsequently used in contingency-table analyses and Firth-penalized logistic regression. The complete wording of the MDAS items, response options, scoring procedure, and categorical interpretation used in the study is provided in Supplementary Material S1.

### 2.6. Statistical Analysis

Statistical analyses were performed using IBM SPSS Statistics (version 25.0; IBM Corp., Armonk, NY, USA) and R software (version 4.6.1; R Foundation for Statistical Computing, Vienna, Austria). SPSS was used for descriptive statistics, non-parametric group comparisons, categorical analyses, and internal-consistency analyses, whereas R was used for Firth-penalized logistic regression. Continuous and ordinal variables were summarized using mean ± standard deviation (SD) and/or median with interquartile range (IQR), as appropriate, whereas categorical variables were presented as frequencies and percentages.

Because the MDAS total and item scores were ordinal or non-normally distributed, non-parametric methods were used for group comparisons. Two-group comparisons were performed using the Mann–Whitney U test, whereas comparisons involving three or more groups were performed using the Kruskal–Wallis test. Significant Kruskal–Wallis tests were followed by pairwise comparisons with Bonferroni adjustment for multiple testing. Differences among the five MDAS item scores were assessed using the Friedman test, followed by pairwise Wilcoxon signed-rank tests with Bonferroni correction (adjusted significance threshold, *p* < 0.005).

Internal consistency of the MDAS was evaluated using Cronbach’s alpha, corrected item–total correlations, and Cronbach’s alpha if individual items were deleted.

Associations between categorical variables and high dental anxiety (MDAS ≥19) were evaluated using Pearson’s chi-square test or exact tests, as appropriate according to expected cell frequencies. The periodontal surgery category (*n* = 3) was excluded from inferential analyses involving primary reason for dental visit because of its extremely small subgroup size.

To examine factors associated with high dental anxiety, multivariable Firth-penalized logistic regression was used because the limited number of high-anxiety events and several zero-event cells resulted in complete or quasi-complete separation for some predictor categories [17]. The full model included gender, age group, education level, dental attendance frequency, and primary reason for dental visit. These variables were selected because they represented the principal demographic and dental-attendance factors addressed by the study objectives, while limiting model complexity in view of the relatively small number of high-anxiety events. Place of residence, previous dental attendance, and history of post-treatment complications were not included in the multivariable model. The periodontal surgery subgroup was excluded from the regression analysis, resulting in an analytic sample of 1002 participants. Adjusted odds ratios (aORs) with 95% confidence intervals (CIs) were reported. The overall effects of multi-category predictors were evaluated using likelihood-ratio tests. All tests were two-sided, and *p* < 0.05 was considered statistically significant unless otherwise specified.

For primary reason for dental visit, root canal treatment was used as the reference category because it contained high-anxiety events and provided a more interpretable comparison than categories with no high-anxiety events. Given the limited number of high-anxiety events and the substantial overlap among several predictors, an additional parsimonious sensitivity model including gender, age group, and dental attendance frequency was fitted. This sensitivity analysis was used to evaluate whether the direction and magnitude of the principal associations were dependent on model specification.

## 3. Results

### 3.1. Participant Characteristics and Dental Anxiety Scores

A total of 1005 participants were included in the analysis, of whom 599 (59.6%) were female and 406 (40.4%) were male (Table 1). Most participants were aged 20–29 years (41.2%), had primary or secondary education (49.4%), lived in a city (71.5%), and had previously visited a dentist (93.4%). The majority reported attending a dentist only when symptomatic (72.1%). The overall median MDAS score was 11 (IQR, 7–15).

MDAS scores differed significantly according to gender, age group, education level, dental attendance frequency, primary reason for dental visit, and history of complications following dental treatment (all *p* < 0.05). Female participants had higher MDAS scores than males [11 (8–15) vs. 10 (7–14), *p* < 0.001]. Significant differences were also observed among age groups (H = 54.302, *p* < 0.001), education levels (H = 168.707, *p* < 0.001), dental attendance frequency groups (H = 78.397, *p* < 0.001), and reasons for dental attendance (H = 40.252, *p* < 0.001).

Bonferroni-adjusted pairwise comparisons showed significant age-group differences between participants aged <20 and 30–39 years, 20–29 and 30–39 years, 20–29 and 40–49 years, 30–39 and 40–49 years, and 40–49 and 50–59 years (all adjusted *p* ≤ 0.002). All three education groups differed significantly from one another (all adjusted *p* < 0.001). Participants attending every 6 months or annually had significantly lower MDAS scores than those attending only when symptomatic (both adjusted *p* < 0.001) and first-time attendees (adjusted *p* = 0.003 and *p* = 0.002, respectively). For the primary reason for dental visit, participants presenting for root canal treatment had significantly higher MDAS scores than those presenting for filling, scaling, or orthodontic treatment (adjusted *p* < 0.001, *p* = 0.001, and *p* < 0.001, respectively). No other pairwise comparisons remained significant after Bonferroni correction.

In contrast, MDAS scores did not differ significantly according to place of residence (*p* = 0.260) or previous dental visit history (*p* = 0.095) (Table 1).

### 3.2. Distribution and Internal Consistency of the MDAS

The mean total MDAS score was 11.17 ± 4.30, with a median of 11 (IQR, 7–15). Among the individual MDAS items, anticipation of a dental visit had the highest mean score (2.74 ± 1.22), followed by local anesthetic injection (2.38 ± 1.24), whereas scaling had the lowest mean score (1.98 ± 1.09).

MDAS item scores differed significantly across the five dental situations (Friedman χ^2^(4) = 624.477, *p* < 0.001). Bonferroni-adjusted Wilcoxon signed-rank tests showed that anxiety related to anticipating a dental visit was significantly higher than anxiety while waiting in the dental clinic (Z = −15.616, *p* < 0.001), dental drilling (Z = −15.565, *p* < 0.001), scaling (Z = −17.068, *p* < 0.001), and local anesthetic injection (Z = −7.620, *p* < 0.001). Anxiety related to local anesthetic injection was also significantly higher than anxiety while waiting in the dental clinic (Z = −8.885, *p* < 0.001), dental drilling (Z = −8.348, *p* < 0.001), and scaling (Z = −8.567, *p* < 0.001). Differences among waiting in the dental clinic, dental drilling, and scaling did not remain significant after Bonferroni correction.

The internal consistency of the five-item MDAS was good (Cronbach’s α = 0.812). Corrected item–total correlations ranged from 0.468 to 0.734, with the strongest item–total association observed for anxiety while waiting in the dental clinic. Cronbach’s α if an individual item was deleted ranged from 0.741 to 0.818, indicating no substantial improvement in internal consistency after removal of any item (Table 2).

### 3.3. Distribution of High Dental Anxiety According to Participant Characteristics

High dental anxiety, defined as an MDAS score ≥ 19, was identified in 49 of 1005 participants (4.9%). The prevalence differed significantly according to gender (*p* < 0.001), age group (*p* < 0.001), education level (*p* < 0.001), frequency of dental attendance (*p* < 0.001), and primary reason for dental visit (*p* < 0.001). High dental anxiety was observed exclusively among women (8.2%), and its prevalence was highest among participants aged 30–39 years (18.3%) and those with a literacy level or lower education (37.8%). Regarding the primary reason for attendance, the highest prevalence was observed among participants seeking prosthetic treatment (24.8%), followed by those undergoing root canal treatment (8.2%). No significant associations were observed for place of residence, previous dental attendance, or history of post-treatment complications (Table 3).

### 3.4. Factors Associated with High Dental Anxiety

Because several predictor categories contained no participants with high dental anxiety, Firth-penalized logistic regression was used to reduce bias related to complete or quasi-complete separation. The periodontal surgery subgroup (*n* = 3) was excluded from the multivariable model because of its very small sample size, resulting in an analytic sample of 1002 participants (Table 4).

In the multivariable model, gender (global *p* < 0.001), age group (*p* = 0.012), dental attendance frequency (*p* = 0.002), and primary reason for dental visit (*p* < 0.001) showed significant overall associations with high dental anxiety, whereas education level was not significantly associated with the outcome after adjustment (global *p* = 0.317).

Female participants had substantially higher odds of high dental anxiety than male participants (aOR = 71.87, 95% CI 6.09–20,128.94; *p* < 0.001). Compared with participants aged < 20 years, those aged 30–39 years had higher odds of high dental anxiety (aOR = 16.36, 95% CI 1.85–244.28; *p* = 0.012), whereas the other age categories were not significantly associated with the outcome. However, these estimates were highly imprecise, particularly for gender, because all 49 high-anxiety events occurred among women and several age categories contained few or no events.

Compared with first-time attendees, participants attending every 6 months (aOR = 0.016, 95% CI 0.0001–0.20; *p* < 0.001) and annually (aOR = 0.017, 95% CI 0.0001–0.60; *p* = 0.022) had significantly lower odds of high dental anxiety. Attendance only when symptomatic was not significantly associated with high dental anxiety after adjustment (aOR = 0.30, 95% CI 0.07–1.27; *p* = 0.100).

Using root canal treatment as the reference category, extraction (aOR = 0.017, 95% CI 0.0001–0.19; *p* < 0.001), filling (aOR = 0.033, 95% CI 0.0002–0.36; *p* = 0.001), and orthodontic treatment (aOR = 0.026, 95% CI 0.0002–0.22; *p* < 0.001) were associated with lower odds of high dental anxiety. Scaling (aOR = 0.34, 95% CI 0.06–1.39; *p* = 0.138) and prosthetic treatment (aOR = 0.43, 95% CI 0.11–1.63; *p* = 0.212) did not differ significantly from root canal treatment. Several confidence intervals remained wide, reflecting sparse and highly clustered high-anxiety events within some predictor strata.

In the parsimonious sensitivity model including gender, age group, and dental attendance frequency, all three predictors showed significant overall associations with high dental anxiety (gender: likelihood-ratio χ^2^ = 41.76, df = 1, *p* < 0.001; age group: χ^2^ = 58.58, df = 4, *p* < 0.001; dental attendance frequency: χ^2^ = 24.74, df = 3, *p* < 0.001). The overall directional pattern was broadly similar to that of the full model, particularly for gender and dental attendance frequency; however, several category-specific estimates, especially those for age, changed substantially and remained imprecise. This indicates that the magnitude of some associations was sensitive to model specification.

## 4. Discussion

The present study evaluated dental anxiety in a large clinical sample using both continuous MDAS scores and the established MDAS ≥19 threshold for high dental anxiety. High dental anxiety was identified in 4.9% of participants, while MDAS scores also varied according to several demographic and dental attendance characteristics. In the multivariable Firth model, gender, age group, dental attendance frequency, and primary reason for dental visit showed significant overall associations with high dental anxiety, whereas educational level was not significantly associated with the outcome after adjustment. A parsimonious sensitivity analysis produced a broadly similar overall pattern, particularly for gender and dental attendance frequency, although the magnitude of several category-specific estimates, especially those for age, was sensitive to model specification. Considering both continuous MDAS scores and the high-anxiety category is important because these approaches reflect related but distinct aspects of the anxiety spectrum, particularly when clinically important high anxiety is relatively uncommon.

The prevalence of high dental anxiety observed in this clinical sample was relatively low compared with several previous reports. A systematic review and meta-analysis demonstrated substantial variation in the prevalence of dental fear and anxiety across populations, partly reflecting differences in instruments, thresholds, and study settings [18]. Recent studies using the MDAS have similarly reported considerable variation in dental anxiety across different populations and clinical settings, including adult samples from Croatia, Greece, and Sri Lanka [4,19,20]. Turkish studies using the MDAS have likewise reported varying levels of dental anxiety [21,22]. Such comparisons should be interpreted cautiously because studies based specifically on the MDAS ≥19 threshold are more directly comparable than those using other instruments or definitions. In addition, the clinic-based sampling frame and the absence of participants aged ≥60 years limit the generalizability of the observed 4.9% frequency, which should not be interpreted as a population prevalence estimate. The relatively low proportion of high dental anxiety in this sample may therefore reflect differences in regional characteristics, clinical setting, dental service-use patterns, and case mix.

Sex was one of the most prominent factors associated with dental anxiety. Women had higher MDAS scores, and all participants meeting the MDAS ≥19 criterion were female. This pattern is consistent with previous studies reporting higher levels of dental anxiety among women [21,22]. However, the absence of high-anxiety events among men produced complete separation and contributed to the very large adjusted odds ratio for female sex [17]. Although Firth-penalized logistic regression provides finite, bias-reduced estimates under separation [17], the resulting effect estimate remained highly imprecise, as reflected by the very wide confidence interval. The association should therefore be interpreted as evidence of a strong directional signal rather than as a precise estimate of effect magnitude.

Age was also associated with dental anxiety, although the pattern was not strictly linear. In the full multivariable model, participants aged 30–39 years had higher odds of high dental anxiety than those aged <20 years, whereas the other age categories were not statistically significant. Previous longitudinal and epidemiological studies have shown that dental anxiety may vary across age groups and may change from adolescence into adulthood [23,24]. Recent findings have also been inconsistent across populations. Some clinical studies have reported significant associations between age and dental anxiety, while others have found no independent age effect. Notably, a recent study of Nepali adults reported the highest mean MDAS score among participants aged 30–39 years, broadly consistent with the age pattern observed in the present sample [4,25,26]. Importantly, age-specific estimates changed substantially in the parsimonious sensitivity model, indicating that the magnitude of these associations was sensitive to model specification. Therefore, the observed age-related differences should be interpreted cautiously, particularly given the small number of high-anxiety events in several age categories.

Educational level differed significantly across unadjusted MDAS analyses but was not independently associated with high dental anxiety in the multivariable Firth model. Previous studies have reported inconsistent associations between education and dental anxiety. Some studies have suggested that lower educational attainment is associated with higher levels of dental anxiety, whereas others have found no significant association between education and anxiety scores. Recent evidence remains similarly mixed, with some studies reporting higher MDAS scores among individuals with lower educational levels, while others have found no significant educational gradient in dental anxiety [7,27,28]. In the present study, the attenuation of the association after adjustment suggests that educational level may overlap with other demographic and dental-care characteristics rather than representing an independent predictor of high dental anxiety.

Dental attendance behavior showed a clear association with dental anxiety. Participants who attended regularly tended to have lower MDAS scores and lower odds of high dental anxiety than those attending only when symptomatic or presenting for a first visit. This finding is consistent with previous evidence linking dental fear with avoidance and irregular attendance [29,30]. However, because the present study was cross-sectional, the direction of this association cannot be determined. Regular attendance may be associated with lower anxiety through repeated tolerable dental experiences, but it is equally plausible that individuals with lower anxiety are more likely to attend regularly. Accordingly, the observed relationship should be interpreted as an association rather than evidence that regular dental attendance reduces dental anxiety.

Primary reason for dental attendance was also associated with high dental anxiety in the multivariable analysis. When root canal treatment was used as the reference category, extraction, filling, and orthodontic treatment were associated with lower odds of high dental anxiety, whereas scaling and prosthetic treatment did not differ significantly from root canal treatment. Previous evidence has identified endodontic treatment, local anesthetic injection, drilling, and other invasive procedures as potentially anxiety-provoking aspects of dental care [31,32]. However, the present procedure-specific estimates should be interpreted cautiously because high-anxiety events were sparse and unevenly distributed across treatment categories. The substantial overlap between visit reason and other participant characteristics also suggests that these category-specific estimates may be sensitive to the composition of the sample rather than reflecting intrinsic differences in the anxiety-provoking potential of individual procedures. Although a history of post-treatment complications was associated with differences in the continuous MDAS score, it was not significantly associated with the dichotomized high-dental-anxiety outcome, likely reflecting the loss of information after dichotomization and the relatively small number of participants meeting the MDAS ≥ 19 threshold.

The MDAS demonstrated good internal consistency in the present sample, supporting its reliability for assessing dental anxiety in this clinical population. Item-level analysis showed that anxiety was not confined to invasive procedures but was already evident in the anticipatory phase, with anticipation of a dental visit producing the highest mean item score [13,21]. Recent psychometric work has also emphasized the distinction between anticipatory and treatment-related components of dental anxiety, supporting the clinical relevance of anxiety that emerges before treatment begins. Procedure-related concerns nevertheless remained important, particularly for local anesthetic injection and other potentially stressful dental procedures [12,33,34]. These findings support the potential value of brief anxiety screening before treatment, as identifying highly anxious patients before the procedure may facilitate tailored communication and anxiety-management strategies.

Several strengths and limitations should be considered. The relatively large sample size, use of a validated Turkish version of the MDAS, and assessment of both continuous anxiety scores and clinically defined high dental anxiety are important strengths. In addition, Firth-penalized logistic regression permitted multivariable estimation in the presence of separation and sparse outcome categories. However, the single-center, clinic-based, and cross-sectional design limits generalizability and precludes causal inference. Individuals with severe dental anxiety may be less likely to seek dental care and therefore may have been underrepresented, introducing potential selection bias. No participants aged ≥ 60 years were represented in the final analytic sample, further limiting the applicability of the findings to older adults. High dental anxiety was uncommon, and several predictor categories contained few or no events; consequently, some adjusted odds ratios were highly imprecise and sensitive to model specification. Penalized regression can reduce estimation bias under separation but cannot eliminate the underlying uncertainty caused by sparse data. The exclusion of individuals with diagnosed psychiatric disorders may also have reduced the observed frequency of dental anxiety and should be considered when interpreting the findings. Finally, potentially relevant factors such as socioeconomic status, previous traumatic dental experiences, pain history, and broader psychological characteristics were not comprehensively assessed.

Overall, these findings indicate that high dental anxiety affected a relatively small but clinically relevant subgroup of participants and was associated with gender, age, dental attendance patterns, and reason for seeking dental care. The consistency of the general pattern across the primary and parsimonious Firth models supports the relevance of these factors, although the magnitude of several category-specific associations remained unstable because of sparse and clustered events. The findings particularly highlight the importance of recognizing anxiety among patients with irregular or symptom-driven attendance and of assessing anxiety before potentially stressful dental procedures. Brief screening with a validated instrument such as the MDAS may help clinicians identify patients who could benefit from individualized communication and anxiety-management strategies. However, these associations should be interpreted within the limitations of the clinic-based, cross-sectional design and should not be regarded as causal or population-level estimates.

## 5. Conclusions

Dental anxiety in this clinical sample was generally low to moderate, although a small subgroup met the threshold for high dental anxiety. Gender, age group, dental attendance frequency, and primary reason for dental visit were associated with high dental anxiety, whereas education level was not independently associated with the outcome after adjustment. Regular dental attendance was associated with lower odds of high dental anxiety, but the cross-sectional design does not allow the direction of this relationship to be determined. Procedure-specific and other category-specific estimates should be interpreted cautiously because several were imprecise and sensitive to sparse and clustered events. Brief anxiety screening and individualized communication may help identify and support patients with greater dental anxiety. These findings should be interpreted as associations rather than causal effects and should not be generalized directly to the broader population.

## Figures and Tables

**Table 1:** Participant characteristics and comparison of Modified Dental Anxiety Scale scores according to demographic and dental characteristics.

Characteristic	*n* (%)	MDAS Score, Median (IQR)	Test Statistic	*p*-Value
Gender			U = 102,974.0	<0.001
Female	599 (59.6)	11 (8–15)		
Male	406 (40.4)	10 (7–14)		
Age group			H = 54.302	<0.001
<20 years	210 (20.9)	10 (8–13)		
20–29 years	414 (41.2)	11 (7–16)		
30–39 years	191 (19.0)	13 (11–15)		
40–49 years	167 (16.6)	9 (6–14)		
50–59 years	23 (2.3)	15 (13–16)		
Education level			H = 168.707	<0.001
Literate or lower	90 (9.0)	15 (15–21)		
Primary/secondary education	496 (49.4)	9 (6–13)		
University or higher	419 (41.7)	12 (9–15)		
Place of residence			H = 2.694	0.260
Village	86 (8.6)	10 (8–13)		
District	200 (19.9)	11 (8–13)		
City	719 (71.5)	11 (7–15)		
Previous dental visit			U = 27,200.0	0.095
No	66 (6.6)	9 (6–17)		
Yes	939 (93.4)	11 (7–15)		
Frequency of dental attendance			H = 78.397	<0.001
First visit	95 (9.5)	11 (9–12)		
Only when symptomatic	725 (72.1)	12 (7–15)		
Every 6 months	161 (16.0)	9 (6–11)		
Annually	24 (2.4)	8 (5–8.75)		
History of complication after dental treatment			U = 54,873.0	0.015
No	859 (85.5)	11 (7–14)		
Yes	146 (14.5)	11.5 (8–16)		
Primary reason for dental visit ^†^			H = 40.252	<0.001
Extraction	240 (23.9)	13 (7–15)		
Filling	156 (15.5)	9 (7–13)		
Root canal treatment	159 (15.8)	12 (10–16)		
Scaling	163 (16.2)	10 (9–11)		
Prosthetic treatment	137 (13.6)	10 (6–18)		
Orthodontic treatment	147 (14.6)	11 (8–12)		

Note: Data are presented as *n* (%) or median (interquartile range). MDAS, Modified Dental Anxiety Scale; U, Mann–Whitney U statistic; H, Kruskal–Wallis H statistic. Mann–Whitney U tests were used for two-group comparisons and Kruskal–Wallis tests for comparisons involving three or more groups. Bold values indicate statistical significance at *p* < 0.05. ^†^ Periodontal surgery (*n* = 3) was excluded from the inferential analysis of primary reason for dental visit because of the very small subgroup size.

**Table 2:** Descriptive and internal consistency characteristics of the Modified Dental Anxiety Scale.

MDAS Item	Mean ± SD	Median (IQR)	Corrected Item–Total Correlation	Cronbach’s α if Item Deleted
Anticipating a dental visit	2.74 ± 1.22	3 (2–3)	0.556	0.790
Waiting in the dental clinic	2.06 ± 1.00	2 (1–3)	0.734	0.741
Dental drilling	2.01 ± 1.13	2 (1–3)	0.674	0.752
Scaling	1.98 ± 1.09	2 (1–3)	0.607	0.773
Local anesthetic injection	2.38 ± 1.24	2 (1–3)	0.468	0.818
MDAS total score	11.17 ± 4.30	11 (7–15)	—	0.812 ^a^

Note: Data are presented as mean ± standard deviation (SD) and median (interquartile range [IQR]), as appropriate. MDAS, Modified Dental Anxiety Scale. ^a^ Overall Cronbach’s α for the five-item MDAS.

**Table 3:** Prevalence of high dental anxiety according to participant characteristics.

Variable	Total, *n*	MDAS ≥ 19, *n* (%)	*p*
Gender			<0.001
Female	599	49 (8.2)	
Male	406	0 (0.0)	
Age group			<0.001
<20 years	210	1 (0.5)	
20–29 years	414	9 (2.2)	
30–39 years	191	35 (18.3)	
40–49 years	167	4 (2.4)	
50–59 years	23	0 (0.0)	
Education level			<0.001
Literate or lower	90	34 (37.8)	
Primary/secondary education	496	6 (1.2)	
University or higher	419	9 (2.1)	
Place of residence			0.771
Village	86	5 (5.8)	
District	200	8 (4.0)	
City	719	36 (5.0)	
Previous dental visit			0.069 ^a^
No	66	0 (0.0)	
Yes	939	49 (5.2)	
Dental attendance frequency			*p* < 0.001 ^a^
First visit	95	4 (4.2)	
Only when symptomatic	725	45 (6.2)	
Every 6 months	161	0 (0.0)	
Annually	24	0 (0.0)	
Post-treatment complication			0.379
No	859	44 (5.1)	
Yes	146	5 (3.4)	
Primary reason for dental visit			<0.001
Extraction	240	0 (0.0)	
Filling	156	0 (0.0)	
Root canal treatment	159	13 (8.2)	
Scaling	163	2 (1.2)	
Prosthetic treatment	137	34 (24.8)	
Orthodontic treatment	147	0 (0.0)	
Periodontal surgery	3	—	—

^a^ Fisher–Freeman–Halton exact test; otherwise Pearson’s chi-square test. Periodontal surgery (*n* = 3) was excluded from inferential analysis.

**Table 4:** Multivariable Firth-Penalized Logistic Regression Analysis of Factors Associated with High Dental Anxiety.

Predictor	β	SE	aOR (95% CI)	*p*
Gender: Female vs. Male	4.275	1.443	71.87 (6.09–20,128.94)	<0.001
Age 20–29 vs. <20	0.843	1.074	2.32 (0.22–48.09)	0.511
Age 30–39 vs. <20	2.795	1.006	16.36 (1.85–244.28)	0.012
Age 40–49 vs. <20	1.088	0.996	2.97 (0.40–40.10)	0.305
Age 50–59 vs. <20	−1.352	1.809	0.26 (0.001–8.42)	0.457
Primary/secondary education vs. Literate/lower	−0.990	0.650	0.37 (0.10–1.50)	0.156
University or higher vs. Literate/lower	−0.304	1.065	0.74 (0.06–20.31)	0.829
Only symptomatic vs. First visit	−1.203	0.657	0.30 (0.07–1.27)	0.100
Every 6 months vs. First visit	−4.117	1.461	0.016 (0.0001–0.20)	<0.001
Annually vs. First visit	−4.047	1.843	0.017 (0.00006–0.60)	0.022
Extraction vs. Root canal	−4.064	1.443	0.017 (0.0001–0.19)	<0.001
Filling vs. Root canal	−3.402	1.361	0.033 (0.0002–0.36)	0.001
Scaling vs. Root canal	−1.069	0.692	0.34 (0.06–1.39)	0.138
Prosthetic vs. Root canal	−0.840	0.647	0.43 (0.11–1.63)	0.212
Orthodontic vs. Root canal	−3.656	1.362	0.026 (0.0002–0.22)	<0.001

aOR, adjusted odds ratio; CI, confidence interval. Odds ratios were estimated using multivariable Firth-penalized logistic regression. The model included gender, age group, education level, dental attendance frequency, and primary reason for dental visit. The periodontal surgery subgroup (*n* = 3) was excluded from the multivariable analysis because of the very small subgroup size; therefore, the regression model included 1002 participants. Reference categories were male gender, age < 20 years, literate or lower education, first dental visit, and root canal treatment. Category-specific estimates should be interpreted cautiously because of sparse and clustered high-anxiety events and several zero-event cells.

## Data Availability

The data presented in this study are available on request from the corresponding author due to ethical reason.

## Supplementary Materials

The following supporting information can be downloaded at: https://www.mdpi.com/article/10.3390/healthcare14193190/s1, Supplementary Material S1: Study Questionnaire.
