# Supplementary material for: Dental Anxiety and Associated Demographic and Dental Attendance Factors: A Cross-Sectional Study Using the Modified Dental Anxiety Scale
Source: Healthcare (Basel). 2026 Sep 28;14(19):3190. doi: 10.3390/healthcare14193190 (PMC13665276; doi:10.3390/healthcare14193190)
Supplement: Supplementary file 1 [file healthcare-14-03190-s001.zip › healthcare-4572135-supplementary.pdf]

Supplementary Material S1. Study Questionnaire

Section A. Demographic and Dental Characteristics

Participants were asked to select the response option that best described their demographic characteristics and dental history.

1. Gender

- Female
- Male

2. Age

- <20 years
- 20–29 years
- 30–39 years
- 40–49 years
- 50–59 years
- ≥60 years

3. Educational status

- Illiterate
- Literate without formal schooling
- Primary school
- Middle school
- High school
- Associate degree
- Bachelor’s degree
- Master’s degree

4. Place of residence

- Village
- District
- City

5. Have you previously visited a dentist?

- Yes
- No

6. Primary reason for the dental visit

- Tooth extraction

- Filling/restorative treatment
- Root canal treatment
- Scaling
- Prosthetic treatment
- Orthodontic treatment
- Periodontal surgical procedure

7. Frequency of dental attendance

- First dental visit
- Only when symptomatic
- Regularly every 6 months
- Regularly once a year

8. Have you previously experienced a complication following dental treatment?

- Yes
- No

Section B. Modified Dental Anxiety Scale (MDAS)

Dental anxiety was evaluated using the five-item Modified Dental Anxiety Scale (MDAS). Participants were asked to select one response for each item. Responses were scored from **1 to 5**, with higher scores indicating greater dental anxiety. The total MDAS score was calculated by summing the five item scores, yielding a possible range of **5–25**.

Item 1. Anticipating a dental visit

If you were going to the dentist tomorrow, how would you feel about it?

1. I would look forward to it as a reasonably enjoyable experience.
2. I would not care one way or the other.
3. I would feel slightly uneasy.
4. I would be afraid that it would be unpleasant and painful.
5. I would be very frightened about what the dentist might do.

Item 2. Waiting in the dental clinic

If you were sitting in the waiting room of a dental clinic, waiting for your turn, how would you feel?

1. Relaxed
2. Slightly uneasy
3. Tense

4. Anxious or distressed
5. Extremely frightened, with physical symptoms such as sweating or nausea

Item 3. Dental drilling

If you were sitting in the dental chair and waiting while the dentist prepared the rotary instruments for treatment, how would you feel?

1. Relaxed
2. Slightly uneasy
3. Tense
4. Anxious or distressed
5. Extremely frightened, with physical symptoms such as sweating or nausea

Item 4. Scaling

If you were sitting in the dental chair and waiting while the dentist prepared the instruments for scaling around your teeth and gums, how would you feel?

1. Relaxed
2. Slightly uneasy
3. Tense
4. Anxious or distressed
5. Extremely frightened, with physical symptoms such as sweating or nausea

Item 5. Local anesthetic injection

If the dentist were about to administer a local anesthetic injection into the gum above one of your upper posterior teeth, how would you feel?

1. Relaxed
2. Slightly uneasy
3. Tense
4. Anxious or distressed
5. Extremely frightened, with physical symptoms such as sweating or nausea

---

Scoring and interpretation

Each MDAS item was scored from **1 to 5**. The total score was calculated as:

MDAS total score = Item 1 + Item 2 + Item 3 + Item 4 + Item 5

Possible total scores ranged from **5 to 25**, with higher scores indicating greater dental anxiety. For the categorical analyses used in the present study:

- **MDAS <19:** no high dental anxiety
- **MDAS ≥19:** high dental anxiety

Supplementary note

For statistical analyses, the original educational categories were collapsed into three groups:

- **Literate or lower:** illiterate + literate without formal schooling
- **Primary/secondary education:** primary school + middle school + high school
- **University or higher:** associate degree + bachelor’s degree + master’s degree

The original questionnaire categories were retained in this supplementary document to reflect the instrument as administered to participants.
